# Issues with Cefiderocol Testing: Comparing Commercial Methods to Broth Microdilution in Iron-Depleted Medium—Analyses of the Performances, ATU, and Trailing Effect According to EUCAST Initial and Revised Interpretation Criteria

**DOI:** 10.3390/diagnostics14202318

**Published:** 2024-10-18

**Authors:** Stefano Stracquadanio, Alice Nicolosi, Andrea Marino, Maddalena Calvo, Stefania Stefani

**Affiliations:** 1 Department of Biomedical and Biotechnological Sciences, University of Catania, 95123 Catania, Italy; s.stracquadanio@unict.it (S.S.); alice.nicolosi@unict.it (A.N.); 2 Unit of Infectious Diseases, Department of Clinical and Experimental Medicine, ARNAS Garibaldi Hospital, University of Catania, 95123 Catania, Italy; andrea.marino@unict.it; 3 U.O.C. Laboratory Analysis Unit, A.O.U. “Policlinico-San Marco”, 95123 Catania, Italy; maddalenacalvo@gmail.com

**Keywords:** cefiderocol, AST, ATU, trailing effect

## Abstract

Background: The rise of multi-drug-resistant Gram-negative bacteria necessitates the development of new antimicrobial agents. Cefiderocol shows promising activity by exploiting bacterial iron transport systems to penetrate the outer membranes of resistant pathogens. Objectives: This study evaluates the efficacy of cefiderocol testing methods and trailing effect impact using a ComASP^®^ Cefiderocol panel, disk diffusion (DD), and MIC test strips (MTS) compared to iron-depleted broth microdilution (ID-BMD). Methods: A total of 131 Gram-negative strains from clinical samples was tested by commercial methods and the gold standard. Results were interpreted as per 2024 and 2023 EUCAST guidelines. Results: ID-BMD revealed high cefiderocol susceptibility among Enterobacterales and *Pseudomonas aeruginosa*, with one *Klebsiella pneumoniae* isolate being resistant. *Acinetobacter baumannii* exhibited higher MIC values, particularly considering trailing effects that complicated MIC readings. ComASP^®^ showed 97% categorical agreement (CA) and 66% essential agreement (EA) with ID-BMD for Enterobacterales but failed to detect the resistant *K. pneumoniae*. DD tests demonstrated variable CA (72% or 93%), and 38% or 34% of strains within the ATU according to EUCAST Breakpoint Tables v13.0 and 14.0, respectively, with major errors only. MTS for *P. aeruginosa* had 100% CA but 44% EA, and often underestimated MIC values. Conclusions: The study emphasizes the need for standardized criteria to address trailing effects and ATU and highlights the discrepancies between testing methods. While cefiderocol resistance remains rare, accurate susceptibility testing is crucial for its effective clinical use. The findings suggest that current commercial tests have limitations, necessitating careful interpretation and potential supplementary testing to guide appropriate antibiotic therapy.

## 1. Introduction

The emergence of multi-drug-resistant (MDR) Gram-negative bacteria represents a challenge in the field of infectious diseases, necessitating the development of novel antimicrobial agents [1]. Cefiderocol (FDC), which is a siderophore cephalosporin, has emerged as a promising candidate in this battle, offering a unique mechanism of action that exploits the bacterial iron transport system to penetrate the outer cell membrane of Gram-negative pathogens, including those resistant to conventional antibiotics, especially (MDR) Enterobacterales, difficult-to-treat *Pseudomonas aeruginosa* (DDT-PA), and carbapenem-resistant *Acinetobacter baumannii* (CRAB) [2,3,4]. This innovative approach enhances cefiderocol’s ability to address the growing concern of antibiotic resistance, making its accurate susceptibility testing crucial for clinical decision-making and patient care [5].

The assessment of cefiderocol’s efficacy against various bacterial strains requires precise and reliable in vitro susceptibility testing methods, which appear to be challenging for microbiologists and clinicians [6]. Among these, the ComASP^®^ Cefiderocol panel stands out as a specialized tool designed for this purpose. The accuracy of susceptibility testing could be pivotal as it informs clinicians about the potential effectiveness of cefiderocol in treating severe infections [7]. This is particularly relevant in hospital settings where rapid and precise identification of antibiotic resistance is critical to effectively manage infections [8].

This study focuses on the evaluation of cefiderocol susceptibility across a collection of clinical Gram-negative bacterial strains using the ComASP^®^ panel alongside other traditional testing methodologies such as disk diffusion (DD) and MIC test strips (MTS^TM^) compared to in-house broth microdilution. By comparing these methods, the study aims to assess the performances of the commercial systems.

Given the evolving landscape of antimicrobial susceptibility testing guidelines and the introduction of new antimicrobial agents like cefiderocol, it is imperative to scrutinize the performance of diagnostic tools such as the ComASP^®^ panel [9]. This examination not only sheds light on the reliability and accuracy of these tools but also contributes to the broader discourse on optimizing antimicrobic susceptibility testing (AST) methodologies to counter the menace of antibiotic resistance effectively. Through this lens, the present study endeavors to provide valuable insights into the efficacy of cefiderocol against a spectrum of Gram-negative pathogens, thereby underpinning its potential role in the antimicrobial armamentarium [10].

## 2. Materials and Methods

### 2.1. Study Sample

The study sample consisted of 131 non-repetitive, clinically significant Gram-negative bacterial strains collected in September–October 2023 from the bloodstream, broncho-alveolar lavage (BAL), and urine of patients hospitalized in different wards at the Policlinico Hospital (Catania, Italy) (Table 1). These strains belonged to ten Enterobacterales species (*Citrobacter freundii* complex, *Citrobacter koseri*, *Enterobacter cloacae*, *Escherichia coli*, *Klebsiella pneumoniae*, *Klebsiella aerogenes*, *Morganella morganii*, *Proteus mirabilis*, and *Serratia marcescens*) and three non-fermentative Gram-negative species (*Acinetobacter baumannii*, *Pseudomonas aeruginosa*, and *Stenotrophomonas maltophilia*) identified by matrix-assisted laser desorption ionization-time of flight (MALDI-TOF) mass spectrometry (Bruker Daltonics, Billerica, MA, USA).

### 2.2. In Vitro Testing

Cefiderocol minimum inhibitory concentrations (MICs) were evaluated by the ComASP^®^ Cefiderocol 0.008–128 mg/L panel (cat. n. 75009, Liofilchem, Roseto degli Abbruzzi, TE, Italy) for all strains according to the manufacturer’s protocol and using *E. coli* ATCC 25922 and *P. aeruginosa* ATCC 27853 as quality controls (expected MIC range 0.06–0.5 mg/L for both strains). *P. aeruginosa* MICs were also performed by MTS^TM^ Cefiderocol 0.016–256 mg/L (cat. n. 920670, Liofilchem, Roseto degli Abbruzzi, TE, Italy), whilst all other species were also tested by DD using Cefiderocol disc 30 μg (cat. n. 9266, Liofilchem, Roseto degli Abbruzzi, TE, Italy) and Mueller Hinton agar (cat. n. CM0337, OXOID, Lowell, MA, USA). All the commercial cefiderocol susceptibility tests were compared to the cefiderocol MIC performed by in-house broth microdilution in iron-depleted cation-adjusted Mueller Hinton broth (ID-BMD) performed as previously published [11,12,13] at a cefiderocol concentration range of 0.03–32 mg/L.

Finally, for 15% of the performed MIC tests, a colony count of the inoculum was performed to avoid biases caused by the inoculum effect.

### 2.3. Interpretation

Cefiderocol susceptibility categorization was interpretated according to 2024 EUCAST guidelines [14,15]. DD results were interpreted according to both EUCAST initial and revised zone diameter criteria. For *A. baumannii* and *S. maltophilia*, a clinical breakpoint for cefiderocol has not yet been established. However, according to EUCAST guidelines and PK/PD studies, isolates with FDC MIC >2 or with a zone diameter <17 mm for *A. baumannii* and <20 mm for *S. maltophilia* should be considered as non-susceptible [14,15,16]. DD tests are prone to uncertain susceptibility evaluation when the inhibition halo diameter is within the area of technical uncertainty (ATU), corresponding to a range of 21–23 mm for Enterobacterales [14]. According to EUCAST, for strains showing a trailing phenomenon, the MIC should be read as the first well in which a reduction of growth corresponding to a button < 1 mm or the presence of light haze/faint turbidity is observed [15].

Performances of ComASP^®^ and MTS^TM^ were evaluated by comparison of their essential agreement (EA) and biases direction using ID-BMD as the gold standard according to ISO [17,18]. DD performances were evaluated only as categorical agreement (CA) with ID-BMD, and the proportion of DD tests falling within the ATU was calculated. Furthermore, a comparison between 2024 and 2023 EUCAST guidelines for DD interpretation was carried out.

With regard to *A. baumannii*, ComASP^®^ performances were evaluated only as EA and biases. Moreover, an analysis of the concordance between DD and ID-BMD was carried out to evaluate the influence of the trailing effect and the reliability of the agar diffusion method. 

Major errors (MEs) and very major errors (VMEs) were calculated as the percentage of false resistant strains and false susceptible strains, respectively, ignoring strains within the ATU. 

### 2.4. Molecular Analyses

The strains with DD results within the ATU and resistant to carbapenems were further tested for the presence of carbapenemase genes (i.e., NDM, IMP, VIM, OXA-48, and KPC) by NG-Test^®^ CARBA-5 (cat. n. NGB-CAR-S23-021, NG-BIOTECH, Guipry-Messac, France).

## 3. Results

### 3.1. Cefiderocol Susceptibility

Performing ComASP^®^ tests, for the two quality control strains—*P. aeruginosa* ATCC 27853 and *E. coli* ATCC 25922—MICs were on target four times (0.125 or 0.25 mg/L) and three times (0.125 or 0.25 mg/L), whilst they were within range (0.5 mg/L) once and twice, respectively. Performing ID-BMD tests, *P. aeruginosa* ATCC 27853 was on target five times (0.125 or 0.25 mg/L), whereas *E. coli* ATCC 25922 was on target four times (0.125 or 0.25 mg/L) and once within range (0.5 mg/L). 

No cefiderocol resistance was observed by ID-BMD among Enterobacterales except for one *K. pneumoniae* isolate. The MIC_90_ and MIC_50_ for Enterobacterales were 1 mg/L and 0.125 mg/L, respectively; *P. aeruginosa* MIC_90_ and MIC_50_ were 0.5 mg/L and 0.125 mg/L; the MIC_90_ and MIC_50_ for *S. maltophilia* were 0.5 mg/L and 0.25 mg/L, though these data are not statistically significant due to the scarce number of isolates (and for this reason they are not reported in the table), while for *A. baumannii*, they were 8 mg/L and 4 mg/L (considering partial growth inhibition given by the trailing effect), and 32 mg/L and 8 mg/L (considering complete growth inhibition by ID-BMD) (Table 2).

ComASP^®^ failed to detect cefiderocol-resistant *K. pneumoniae*, and all the Enterobacterales and *P. aeruginosa* strains were susceptible to cefiderocol. The MIC_90_ and MIC_50_ were 0.5 mg/L and 0.25 mg/L, and 0.25 mg/L and 0.125 mg/L for Enterobacterales and *P. aeruginosa*, respectively. *S. maltophilia* MIC_90_ and MIC_50_ were 0.5 mg/L and 0.25 Mg/L, whilst *A. baumannii* MIC_50_ and MIC_90_ were influenced by the trailing effect and were equal to 2 mg/L and 1 mg/L when ignoring the trailing effect and 128 mg/L and 4 mg/L when considering it (Table 2). 

Finally, MTS for *P. aeruginosa* gave a cefiderocol MIC_90_ of 0.38 mg/L and a MIC_50_ of 0.064 mg/L (Table 2). The MTS result for *P. aeruginosa* ATCC 27853 was 0.25 mg/L, falling within the EUCAST range of 0.06–0.5 mg/L.

### 3.2. ComASP^®^, DD, and MTS Performance Evaluation

**Figure 1:**
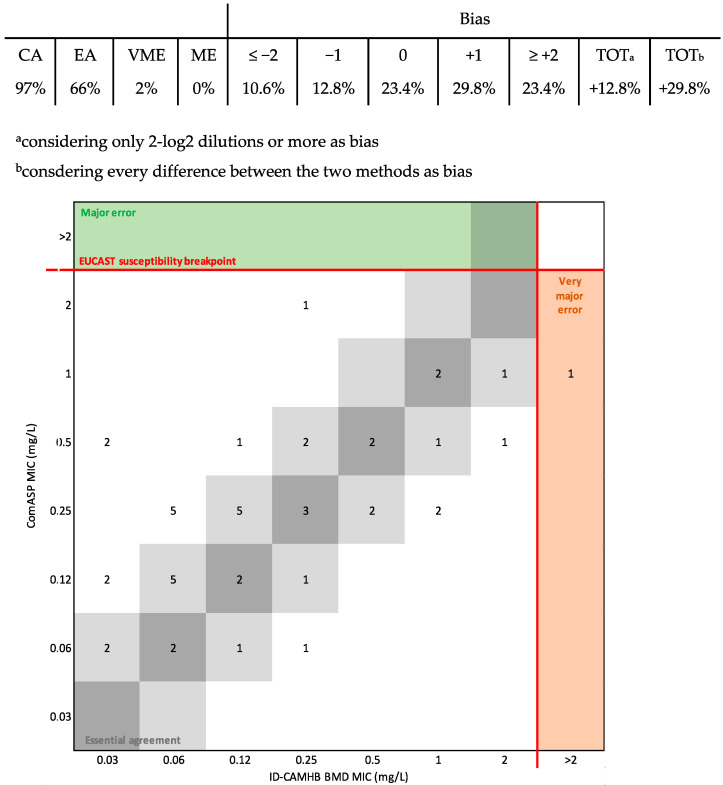
Performances of ComASP^®^ compared to ID-BMD for Enterobacterales. The number of strains with a MIC corresponding to the broth microdilution method and 1-log2 dilution are highlighted in dark and light grey areas, respectively.

**Figure 2:**
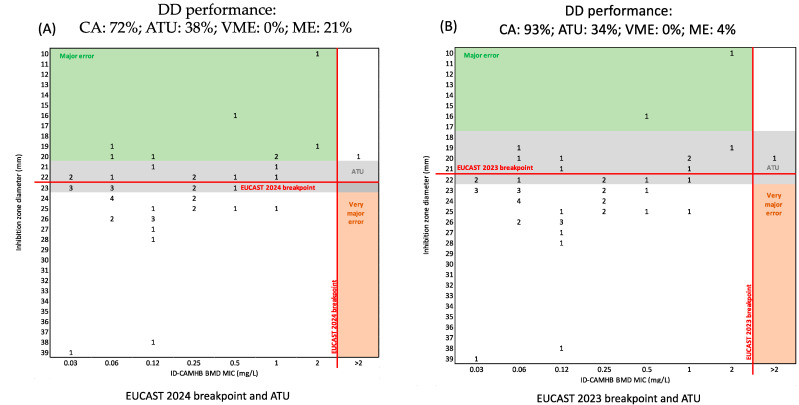
Comparison between DD and ID-BMD MIC for Enterobacterales according to EUCAST initial (**B**) and revised (**A**) zone diameter criteria.

The CA and EA between ComASP^®^ and ID-BMD were 97% and 66%, respectively, for Enterobacterales, with +12.8%, considering only 2-log2 dilutions or more as bias, and +29.8% considering every difference between the two methods as bias. Furthermore, ComASP^®^ displayed 2% of VMEs (Figure 1). No major errors were detected. DD showed 72% CA with new EUCAST breakpoints (Figure 2A) and 93% with old guidelines (Figure 2B), with 38% and 34% of strains within the ATU, respectively, and 21% or 4% of MEs (no VMEs detected). ATU strains were not categorized.

**Figure 3:**
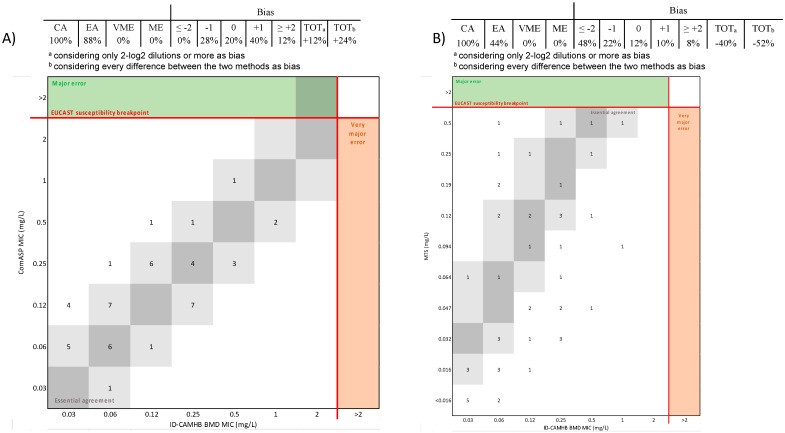
Performances of ComASP^®^ (**A**) and MST (**B**) compared to ID-BMD for *P. aeruginosa*. The number of strains with a MIC corresponding to the broth microdilution method and 1-log2 dilution are highlighted in dark and light grey areas, respectively.

For *P. aeruginosa*, the CA was 100%, with an EA of 88% and 44% between ID-BMD and ComASP^®^ or MTS with a bias of +12% and −40%, respectively, considering only differences of 2-log2 dilutions or more as bias, and +24% and −52%, respectively, treating every discrepancy between the two methods as bias (Figure 3A,B).

**Figure 4:**
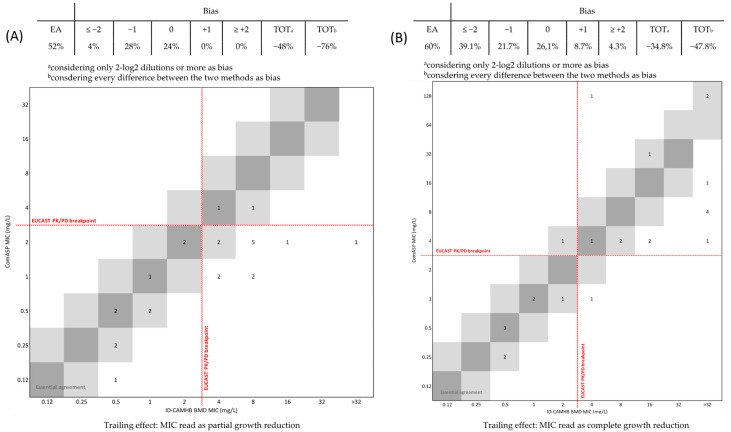
Performances of ComASP^®^ compared to ID-BMD and the impact of the trailing effect for *A. baumannii* reading the MIC as partial (**A**) or complete (**B**) growth reduction. The number of strains with a MIC corresponding to the broth microdilution method and 1-log2 dilution are highlighted in dark and light grey areas, respectively.

The EA for *A. baumannii* was 52% (with −48% and −76% biases, taking into account only differences of 2-log2 dilutions or greater as bias or viewing any discrepancy between the two methods as bias, respectively) when ignoring the trailing effect (Figure 4A), and 60% (with −34.8%, considering only differences of 2-log2 dilutions or more as bias, and −47.8% biases, recognizing every variation between the two methods as potential bias) when considering complete growth inhibition (Figure 4B).

### 3.3. Trailing Effect

A trailing effect was observed with ComASP^®^ when testing one *P. aeruginosa*, four *P. mirabilis,* one *K. pneumoniae*, and 14 out of 25 *A. baumannii* strains. Only *K. pneumoniae* categorization may have been affected by the trailing effect as the breakpoint value was within the trailing range, whilst *P. aeruginosa* and all the *P. mirabilis* were susceptible when reading both partial and complete inhibition.

The ID-BMD showed the same effect in only 12 out of the 14 *A. baumannii* strains and no other species exhibited a trailing effect. ID-BMD evaluation of the *K. pneumoniae* strain presenting a trailing effect (1–4 mg/L) gave a value of 2 mg/L, which was in concordance with the ComASP^®^ categorization and discordant to the DD result of 10 mm.

**Figure 5:**
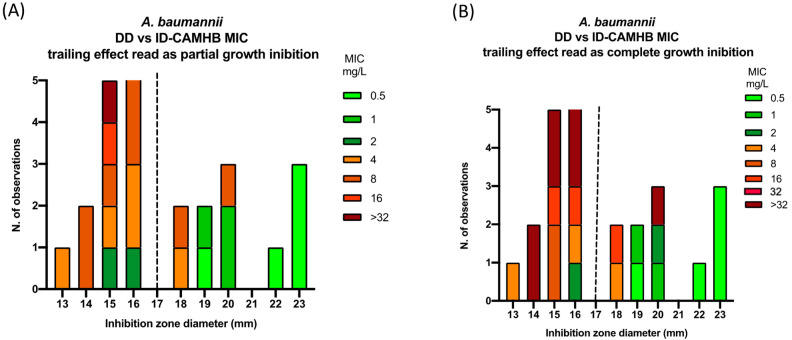
Concordance between DD and ID-BMD and the impact of the trailing effect for *A. baumannii* reading the MIC as partial (**A**) or complete (**B**) growth inhibition. A zone diameter of ≥17 mm corresponds to a MIC value below the PK/PD breakpoint of S ≤ 2 mg/L^15^.

Considering the number (12) and width (1–>32 mg/L) of the trailing effect reported for *A. baumannii*, we further investigated the trailing effect on DD and ID-BMD concordance as reported in Figure 5A,B. Reading the MIC as the reduction in visible growth (ignoring the trailing effect), two strains with an inhibition halo <17 mm had MIC values of 2 mg/L and three strains with an inhibition halo 17 mm had MIC values of 4–8 mg/L. Reading the MIC as complete growth inhibition (considering the trailing effect), one *A. baumannii* strain with an inhibition halo <17 mm had a MIC value of 2 mg/L and three strains with an inhibition halo >17 mm had MIC values of 4 mg/L, 16 mg/L, and >32 mg/L.

### 3.4. ATU Analyses and Comparison Between Old and New Breakpoints

A total of 18 out of 47 (38%) Enterobacterales could not be categorized by DD as their inhibition halo diameters were inside the ATU. In particular, one *C. freundii* complex, two *C. koseri*, one *E. cloacae*, two *P. mirabilis*, one *E. coli*, three *M. morganii,* five *K. pneumoniae*, and three *K. aerogenes* had inhibition zone diameters within 21–23 mm and were susceptible according to both ComASP^®^ and ID-BMD (Figure 2A). Half of the samples inside the ATU had an inhibition halo of 23 mm, which is the upper limit of the ATU range, whilst nine strains had inhibition halos of 21–22 mm.

The DD breakpoint as well as the ATU range have been recently modified by EUCAST, and our results were analyzed according to the new values. Nonetheless, a brief report of the analyses performed considering the previous values (susceptibility breakpoint ≥ 22 mm and ATU 18–22 mm) could provide interesting insights.

Considering the old EUCAST indications, 17 Enterobacterales (34%) could not be categorized by DD as their inhibition zone diameters were within the ATU. Three *K. pneumoniae,* one *M. morganii,* one *E. coli*, and two *P. mirabilis* were inside the ATU with the 2023 EUCAST guidelines instead of being categorized as resistant, while nine isolates currently in the ATU were categorized as susceptible according to the older ATU and breakpoint values.

Of note, all strains (7) that left the ATU with the new breakpoints were resistant by DD but susceptible by ID-BMD (Figure 2A,B). Of these, only one *K. pneumoniae* was resistant by ID-BMD.

### 3.5. Carbapenemase Genes

Among the strains within the ATU, only five *K. pneumoniae* and one *E. coli* were carbapenem-resistant. All of them harbored the KPC gene according to NG-Test^®^ CARBA-5.

## 4. Discussion

The emergence of multi-drug-resistant (MDR) Gram-negative bacteria has driven the need for new and effective antimicrobial agents. Cefiderocol, which is a siderophore cephalosporin, represents a promising solution by leveraging the bacterial iron transport system to penetrate the outer membranes of Gram-negative bacteria, including MDR Enterobacterales, DTT-PA, and CRAB. Accurate susceptibility testing is critical for guiding effective clinical use of cefiderocol.

Although broth microdilution evaluation of the MIC is generally indicated as the gold standard for testing cefiderocol susceptibility, EUCAST guidelines seem to encourage the use of DD in a hospital setting as there is currently no commercially available approved test for cefiderocol MIC determination [19]. To date, the main available methods—i.e., ComASP^®^, DD, and strips—have some limitations and their evaluation is still ongoing as is the definition of cefiderocol clinical breakpoints.

According to our findings, cefiderocol resistance is still uncommon as also reported by Bianco et al. [8]; in fact, our study demonstrated high susceptibility of various Gram-negative pathogens to cefiderocol, with the exception of one *K. pneumoniae* isolate that was resistant to the antibiotic as determined by ID-BMD. MIC_50_ and MIC_90_ values for Enterobacterales and *P. aeruginosa* were notably low, indicating strong efficacy. In contrast, *A. baumannii* exhibited higher MIC values, especially when trailing effects were considered, suggesting potential challenges in treating infections caused by this pathogen with cefiderocol and difficulties in testing the actual MIC values of cefiderocol for *A. baumannii.*

With regard to commercial tests, ComASP^®^ failed to detect the cefiderocol-resistant *K. pneumoniae*, raising concerns about its sensitivity. Nonetheless, the VME rate was lower than that found by Emeraud and colleagues [20] who, however, had an overall higher number of cefiderocol-resistant isolates. Furthermore, ComASP^®^ showed a high percentage of high biases for Enterobacterales and *P. aeruginosa* compared to ID-BMD, possibly overestimating their actual MIC. Nevertheless, the percentage of bias was acceptable, whether considering only the differences of 2-log2 dilutions or higher as bias or taking into consideration any discrepancy between the two methods as bias.

Trailing effects, especially with the ComASP^®^ panel, were observed primarily in *A. baumannii*, affecting MIC readings. The trailing phenomenon, which results in incomplete bacterial inhibition at certain cefiderocol concentrations, led to higher MIC values when complete inhibition was considered. This effect was less pronounced in other species. Consequently, there is a need for standardized criteria to address trailing effects to avoid misclassification of resistance, especially in critical clinical settings.

Overall, despite a good CA between ComASP^®^ and the gold standard method, the former was unacceptable according to ISO [17,18] due to the low percentage of EA.

Despite a 100% CA—probably given by *P. aeruginosa* high susceptibility to cefiderocol—MTS comparison with ID-BMD for *P. aeruginosa* often gave lower MIC values (44% EA, and −40% and −52% of biases, considering only differences of 2-log2 dilutions or more as bias, while viewing any difference between the two methods as a form of bias, respectively), indicating that the strips underestimate the actual cefiderocol MIC in this species as previously found [5,21]. Surprisingly, the MTS result for *P. aeruginosa* ATCC 27853 fell within the EUCAST range for FDC. However, the percentage of biases was unacceptable in any condition.

Fortunately, DD analyses seem to be only prone to major errors, and no very major errors were detected, possibly due to the lack of actual cefiderocol-resistant strains in our sample. It demonstrated a variable CA with ID-BMD [22], which differed depending on whether the 2023 or 2024 EUCAST breakpoints were applied.

Eighteen Enterobacterales could not be categorized as their inhibition zone diameters fell within the ATU. As per EUCAST guidelines, if these strains were categorized ignoring the ATU due to the impossibility of running confirmation tests, half of them would be susceptible and half resistant to cefiderocol. When tested with ID-BMD, all these strains proved susceptible to cefiderocol, confirming the need to perform other tests to solve the ATU before reporting cefiderocol resistance. Eventually, categorizing the strains in the ATU will lead to an increase in false resistant results. EUCAST has recently updated the breakpoints of the DD test and the ATU ranges for cefiderocol testing. Interestingly, if the same results were analyzed using the former values, the CA between DD and ID-BMD would significantly increase to 93% and the ME rate would be reduced by almost 1/5. This is probably due to the larger ATU range that incorporates more of the false resistant strains obtained with the new ATU and is also caused by the presence of nine true susceptible strains that leave the ATU. This underscores the dynamic nature of susceptibility standards and the necessity for continual updates to reflect current resistance patterns.

Finally, with regard to Enterobacterales within the ATU, genetic analyses revealed the presence of KPC genes only in *K. pneumoniae* and *E. coli* strains. No other carbapenemase genes were found and no carbapenemases were detected in *K. aerogenes* strains despite their uncertain categorization with DD. The presence of carbapenemase genes could be responsible for the majority of the strains falling within the ATU.

Due to its multi-drug-resistant profile, *A. baumannii* is the species most deserving the use of new antibiotics, as well as being the one showing most cefiderocol non-susceptible strains and the highest MIC values obtained with both the ComASP^®^ panel and ID-BMD assay. Interestingly, ComASP^®^ seems to underestimate the high resistance of *A. baumannii*, which is in agreement with what was suggested by Kolesnik-Goldman and colleagues [23], and consequently, the categorical agreement between ComASP^®^ and ID-BMD is very low. The Achilles’ heel of cefiderocol testing, especially for *A. baumannii*, is represented by the interpretation of the trailing effect. At the time of this manuscript being written, both EUCAST and FDA guidelines suggest considering the presence of bacterial growth with a button <1 mm as the MIC value [15,24], but in our findings, reading the MIC as complete growth inhibition would increase the concordance rate between the methods. According to our results, this reading procedure slightly increases the concordance between ComASP^®^ and ID-BMD as well as the actual correlation between DD and ID-BMD. In support of this, the percentage of bias would also become acceptable and the lowest reported in our data when reading the MIC as complete growth inhibition and considering only differences of 2-log2 dilutions or higher. On the other hand, when reading at partial inhibition, the percentage of bias was unacceptable in every case.

Although a small number of *A. baumannii* were tested, it would seem that a DD breakpoint of 18 mm may be more appropriate for the currently suggested PK/PD value of 17 mm, which is considered equivalent to a MIC breakpoint of 2 mg/L.

The use of antibiotics should be regulated by their susceptibility testing to preserve their activity. Antibiotic susceptibility testing for new molecules deserves more attention due to the lack of laboratory and clinical experience, and this is even more true for cefiderocol due to its particular testing conditions.

### Study Limitations and Strengths

Although the study includes 131 Gram-negative strains, the sample size may still be considered small, particularly for drawing definitive conclusions about the performance of different testing methods across all species. The limited number of isolates, especially for species like *S. maltophilia*, and the scarceness of cefiderocol-resistant strains may reduce the generalizability of the findings.

The study is geographically limited as the bacterial strains were collected from a single hospital in Italy. While this provides valuable data for that specific region, it may limit the applicability of the findings to other geographic areas where different resistance patterns and bacterial strains might be present.

The study indicates that ComASP^®^ and MIC test strips may either overestimate or underestimate cefiderocol MIC values for certain species. While this finding is critical, it highlights the inherent limitations of the commercial testing methods used in the study and raises concerns about potential biases in the susceptibility data reported. Nonetheless, cefiderocol represents a promising tool in combating these resistant pathogens. The study contributes to the growing body of literature focused on improving antimicrobic susceptibility testing (AST) for this new antibiotic, which is crucial in clinical decision-making.

Unlike most of the studies currently available in the literature, this study adheres to updated 2024 EUCAST guidelines, ensuring that the findings are relevant to current clinical practices. Additionally, the comparison of old and new EUCAST breakpoints for disk diffusion testing highlights the evolving nature of susceptibility standards and the impact of these updates on resistance categorization.

Finally, the study’s investigation of the trailing effect, particularly in A. baumannii, and the ATU provides valuable insights into the interpretation challenges posed by these phenomena in susceptibility testing. The findings emphasize the need for standardized criteria to account for trailing effects, which is crucial for the accurate classification of resistance, and highlight how ATU deserves more attention as its range may affect the choice of using cefiderocol.

## Figures and Tables

**Table 1:** Study sample.

Number Tested	Species
2	*Citrobacter freundii* complex
3	*Citrobacter koseri*
5	*Enterobacter cloacae*
10	*Escherichia coli*
4	*Klebsiella aerogenes*
11	*Klebsiella pneumoniae*
4	*Morganella morganii*
6	*Proteus mirabilis*
2	*Serratia marcescens*
25	*Acinetobacter baumannii*
50	*Pseudomonas aeruginosa*
9	*Stenotrophomonas maltophilia*
131	TOT

**Table 2:** Cefiderocol MIC range, MIC_50_, and MIC_90_ according to the different tested methods.

	ID-BMD			ComASP^®^			MTS		
*Species*	MIC Range [mg/L]	MIC_50_ [mg/L]	MIC_90_ [mg/L]	MIC Range [mg/L]	MIC_50_ [mg/L]	MIC_90_ [mg/L]	MIC Range [mg/L]	MIC_50_ [mg/L]	MIC_90_ [mg/L]
***Enterobacterales***	0.03–4	0.125	1	0.06–2	0.25	0.5			
*C. freundii complex*	0.06			0.06–0.125					
*C. koseri*	0.03–0.06			0.125–0.25					
*E. cloacae*	0.06–0.25			0.125–0.25					
*E. coli*	0.06–1			0.06–0.5					
*K. aerogenes*	0.03–1			0.125–2					
*K. pneumoniae*	0.25–4			0.25–1					
*M. morganii*	0.03–0.06			0.125–0.5					
*P. mirabilis*	0.03–0.125			0.06–0.25					
*S. marcescens*	0.03–0.125			0.06–0.25					
***A. baumannii* (partial growth inhibition)**	0.5–>32	4	8	0.125–4	1	2			
***A. baumannii* (complete growth inhibition)**	0.5–>32	8	32	0.25–128	4	128			
***P. aeruginosa***	0.03–1	0.125	0.5	0.03–1	0.125	0.25	<0.016–0.5	0.064	0.38

## Data Availability

The original contributions presented in the study are included in the article, further inquiries can be directed to the corresponding author.

## List of Abbreviations

AST	antimicrobic susceptibility testing
ATCC	American type culture collection
ATU	area of technical uncertainty
BAL	broncho-alveolar lavage
CA	categorical agreement
CRAB	carbapenem-resistant *Acinetobacter baumannii*
DD	disk diffusion
DDT-PA	difficult-to-treat *Pseudomonas aeruginosa*
EA	essential agreement
EUCAST	European committee on antimicrobial susceptibility testing
FDA	food and drug administration
FDC	cefiderocol
ID-BMD	broth microdilution in iron-depleted cation-adjusted Mueller Hinton broth
IMP	imipenemase
KPC	Klebsiella pneumoniae carbapenemase
MALDI-TOF	matrix-assisted laser desorption ionization-time of flight
MDR	multi-drug-resistant
ME	major error
MIC	minimum inhibitory concentration
MTS	MIC test strip
NDM	New-Delhi metallo-β-lactamase
PK/PD	pharmacokinetic/pharmacodynamic
VIM	Verona integron–encoded metallo-β-lactamase
VME	very major error
